# A framework of biomarkers for renal aging: a consensus statement by the Aging Biomarker Consortium

**DOI:** 10.1093/lifemedi/lnag026

**Published:** 2026-07-13

**Authors:** Aging Biomarker Consortium, Xiaoyan Zhang, Anqun Chen, Linli Lv, Liang Ma, Yan Zheng, Beibei Ma, Ziying Zhong, Yaobin Jing, Aiming Qiu, Yunxia Shao, Weiqi Zhang, Jun Cen, Lili Zhou, Wei Chen, Chun Zhang, Jian Li, Jing Chen, Baoxue Yang, Youhua Liu, Jing Qu, Fan Yi, Jinghong Zhao, Bi-Cheng Liu, Xiong Zhong Ruan, Limin Lu, Feng Zheng, Guang-Hui Liu, Gang Pei, Jing Nie, Youfei Guan

**Affiliations:** Kidney Health Institute, East China Normal University, Shanghai 200241, China; Department of Nephrology, The First Affiliated Hospital of Chongqing Medical University, Chongqing 400016, China; Institute of Nephrology Zhongda Hospital, Jiangsu Provincial Key Laboratory of Renal Injury and Repair, Southeast University School of Medicine, Nanjing 210009, China; Department of Nephrology, Institute of Kidney Diseases, West China Hospital of Sichuan University, Chengdu 610041, China; State Key Laboratory of Genetics and Development of Complex Phenotypes, Fudan University, Shanghai 200433, China; Human Phenome Institute, Fudan University, Shanghai 200433, China; Kidney Health Institute, East China Normal University, Shanghai 200241, China; Biobank of Peking University First Hospital, Department of Clinical Medical Research, Peking University First Hospital, Beijing 100034, China; State Key Laboratory of Vascular Homeostasis and Remodeling, Peking University Health Science Center, Peking University, Beijing 100034, China; Key Laboratory of Reproductive Health Diseases Research and Translation of Ministry of Education, International Center for Aging and Cancer, Hainan Medical University, Haikou 571199, China; The Fifth People’s Hospital of Wujiang Area, Suzhou 215200, China; Second People’s Hospital of Wuhu, Wuhu 241000, China; College of Life Sciences, Anhui Normal University, Wuhu 241002, China; China National Center for Bioinformation and Beijing Institute of Genomics, Chinese Academy of Sciences, Beijing 100101, China; Shanghai Jian Gong Hospital, Jian Gong Hospital Clinical Research Center, East China Normal University, Shanghai 200241, China; Division of Nephrology, Nanfang Hospital, Southern Medical University, Guangzhou 510515, China; National Clinical Research Center for Kidney Disease, Guangzhou 510515, China; Department of Nephrology, The First Affiliated Hospital, Sun Yat-sen University, Guangzhou 510080, China; NHC Key Laboratory of Clinical Nephrology (Sun Yat-sen University) and Guangdong Provincial Key Laboratory of Nephrology, Guangzhou 510080, China; Department of Nephrology, Union Hospital, Tongji Medical College, Huazhong University of Science and Technology, Wuhan 430022, China; The Key Laboratory of Geriatrics, Beijing Institute of Geriatrics, Institute of Geriatric Medicine, Chinese Academy of Medical Sciences, Beijing Hospital/National Center of Gerontology of National Health Commission, Beijing 100730, China; Division of Nephrology, Huashan Hospital, Fudan University, Shanghai 200040, China; National Clinical Research Center for Aging and Medicine, Huashan Hospital, Fudan University, Shanghai 200040, China; Department of Pharmacology, School of Basic Medical Sciences, Peking University, Beijing 100191, China; Division of Nephrology, Nanfang Hospital, Southern Medical University, Guangzhou 510515, China; State Key Laboratory of Organ Regeneration and Reconstruction, Institute of Zoology, Chinese Academy of Sciences, Beijing 100101, China; Department of Pharmacology, Key Laboratory of Infection, Immunity and prevention of Shandong Province, School of Basic Medical Sciences, Shandong University, Jinan 250012, China; Department of Nephrology, Xinqiao Hospital, Army Medical University, Chongqing 400037, China; Institute of Nephrology, Zhong Da Hospital, Southeast University School of Medicine, Nanjing 210009, China; Centre for Lipid Research and Chongqing Key Laboratory of Metabolism on Lipid and Glucose, The Second Affiliated Hospital, Chongqing Medical University, Chongqing 400010, China; Department of Physiology and Pathophysiology, Shanghai Medical School, Fudan University, Shanghai 200032, China; Kidney Health Institute, East China Normal University, Shanghai 200241, China; State Key Laboratory of Organ Regeneration and Reconstruction, Institute of Zoology, Chinese Academy of Sciences, Beijing 100101, China; Collaborative Innovation Center for Brain Science, School of Life Science and Technology, Tongji University, Shanghai 200092, China; Biobank of Peking University First Hospital, Department of Clinical Medical Research, Peking University First Hospital, Beijing 100034, China; State Key Laboratory of Vascular Homeostasis and Remodeling, Peking University Health Science Center, Peking University, Beijing 100034, China; Key Laboratory of Cellular Physiology of the Ministry of Education of China, Department of Physiology, Shanxi Medical University, Taiyuan 030001, China; Advanced Institute for Medical Sciences, Dalian Medical University, Dalian 116044, China

**Keywords:** renal aging, renal aging, renal aging, Aging Biomarker Consortium

## Abstract

Renal aging involves structural and functional kidney decline (reduced size/nephrons, glomerulosclerosis, tubular atrophy, functional loss) and is an independent risk factor for kidney and systemic degenerative diseases. On 16 May 2025, the Aging Biomarker Consortium convened an expert consensus meeting in Shanghai, proposing a multidimensional biomarker framework: functional (estimated glomerular filtration rate, renal blood flow), structural (renal volume loss), and humoral (Klotho, N-terminal Pro-B-type natriuretic peptide, senescence-associated secretory phenotype factors). The consensus also supports machine-learning models for biological age assessment and calls for multi-center cohorts and translational collaboration to improve elderly kidney health.

## Introduction

The kidney performs three essential functions: waste excretion, fluid/acid-base balance, and endocrine activity. Renal aging involves structural changes (reduced size, nephron loss, glomerulosclerosis, tubular atrophy) and functional decline (reduced filtration rate and tubular dysfunction). The aging kidney exhibits increased susceptibility to injurious factors and is an independent risk factor for kidney diseases [1]. In China, 57.7% of hospitalized acute kidney injury (AKI) patients are aged over 60 years, and chronic kidney disease (CKD) prevalence among the elderly ranges from 13.2% to 24.2%. Renal aging is also linked to neurological, cardiovascular, and metabolic diseases [2, 3]. Thus, identifying early biomarkers of renal aging is crucial for prevention and intervention. The Aging Biomarker Consortium (ABC) aims to establish standardized frameworks for evaluating biological aging across multiple organ systems [4–15]. On 16 May 2025, the ABC convened an expert seminar and formed a consensus recommending biomarkers that reflect renal aging, aiming to assess the degree and rate of renal aging and predict associated disease risks.

## Methods

We conducted a literature search (databases: MEDLINE, PubMed, Cochrane Library, others) for studies published before October 2024. ABC members drafted a consensus document on renal aging biomarkers, followed by multiple online revision rounds. On 16 May 2025, an expert review meeting finalized the consensus. All recommendations were reviewed by ABC members. This consensus adopts internationally standardized methods for denoting recommendation categories and evidence levels, as detailed in Table 1.

**Table 1.** Classification and definitions of evidence levels and recommendation categories.

Class (strength) of recommendation	Level (quality) of evidence
**Class I (strong) benefit ≫> risk**	Level A
**Suggested phrases for writing recommendation** **Recommended/indicated** **Evidence and/or general agreement that a given treatment or procedure is beneficial, useful and effective**	Data derived from multiple randomized clinical trials or meta-analysis
**Class IIa (moderate) benefit ≫ risk**	Level B
**Suggested phrases for writing recommendation** **Should be considered** **Weight of evidence/opinion is in favor of usefulness/efficacy**	Data derived from a single randomized clinical trial or large non-randomized studies
**Class IIb (weak) benefit ≥ risk**	Level C
**Suggested phrases for writing recommendation** **May be considered** **Usefulness/efficacy is less well established by evidence/opinion**	Consensus of expert opinion, and/or small studies, retrospective studies, registries
**Class III (strong) risk > benefit**	Note: COR and LOE are determined independently (any COR may be paired with any LOE). COR, class of recommendation; LOE, level of evidence.
**Suggested phrases for writing recommendation** **Not recommended** **Evidence/general agreement that the given treatment/procedure is not useful/effective and sometimes may be harmful**	

## Classification and application of biomarkers for renal aging

**Figure 1.**
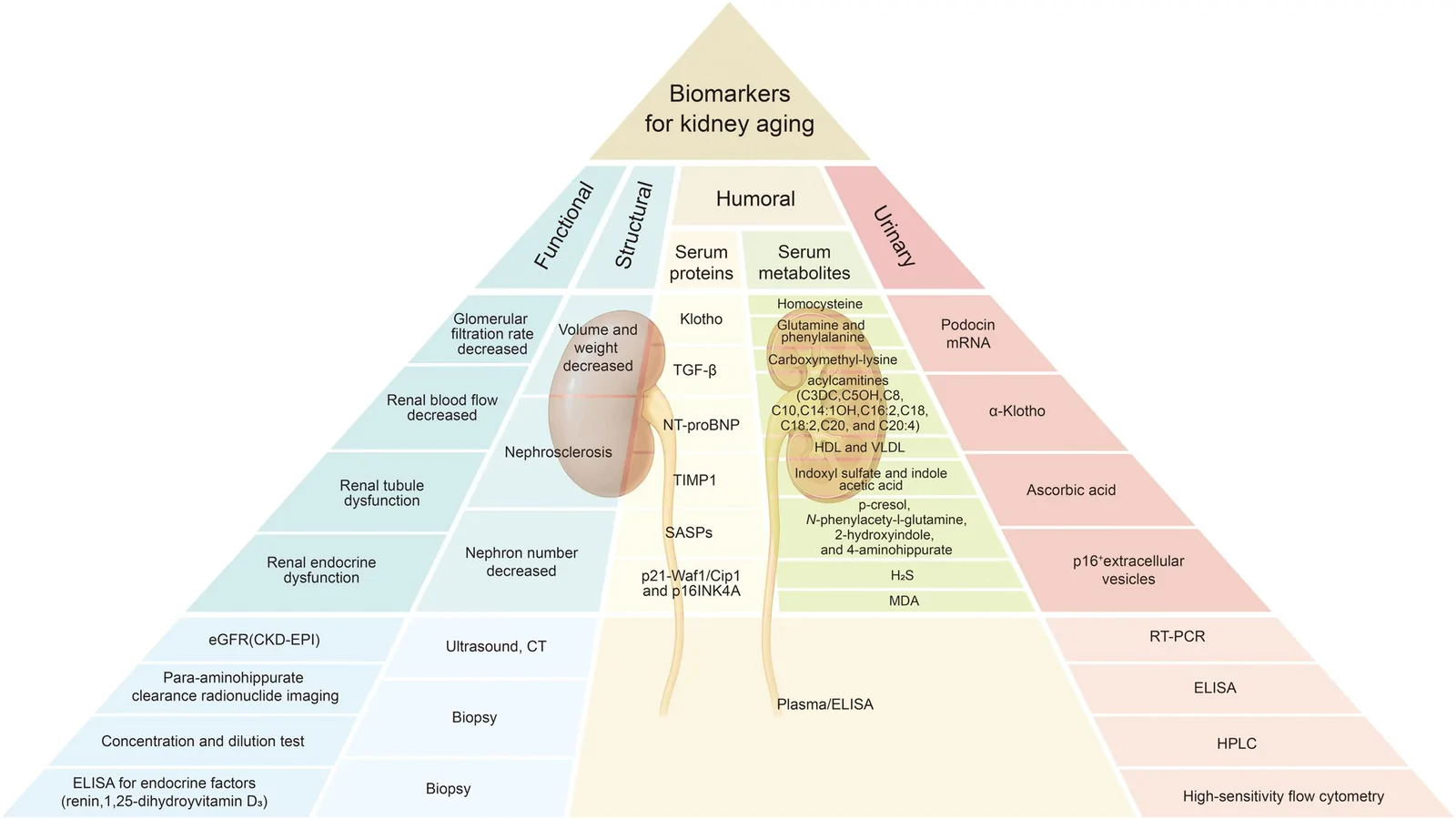
Summary of proposed recommended markers of renal aging

Renal aging involves extensive changes across molecular to systemic levels. Biomarkers of renal aging accurately reflect the kidney’s biological age, structure, and function, and can be used to assess aging degree and evaluate interventions. Based on clinical feasibility, this consensus categorizes biomarkers into three dimensions: renal function, renal structure, and body fluids (Fig. 1).

The proposed framework for the assessment of renal aging consists of three dimensions: functional, imaging, and body fluid biomarkers. A wide range of changes occurring at all levels of the kidney during the aging process are covered.

## Functional characteristics and assessment of renal aging

### Decline in glomerular filtration rate

Glomerular filtration rate (GFR) is a primary indicator of kidney function, reflecting plasma volume filtered by functional nephrons per unit time. In healthy adults, GFR ranges from 100–125 mL/min/1.73 m^2^, estimated based on sex, ethnicity, and age [16, 17]. A study of 365 potential kidney donors using iothalamate clearance showed an age-related GFR decline: from 129 mL/min at age 20, decreasing by 4.6 mL/min per decade in males, and from 123 mL/min at age 20, decreasing by 7.1 mL/min per decade in females [18]. A Cleveland Clinic study over 30 years using 125I-iothalamate in 1057 donors established a normal corrected GFR of 107.6 ± 16.8 mL/min/1.73 m^2^ in Caucasians; donors > 45 years had lower GFR than those <45 years (101.5 ± 16.6 vs. 109.9 ± 16.4 mL/min/1.73 m^2^, *P* < 0.001) [19]. A study in healthy Chinese adults reported average GFR for those under 50 years as approximately 104.0 mL/min/1.73 m^2^ (95% CI: 71.0–137.0) in males and 110.1 mL/min/1.73 m^2^ (95% CI: 75.2–144.9) in females; for those over 60 years, average GFR was 76.1 mL/min/1.73 m^2^ (95% CI: 45.5–106.7) [20].

In 1950, Davis et al. reported that GFR in healthy adults begins to decline from age 30, with a 46% decrease by age 90. A Baltimore longitudinal aging study (1958–1981) of 254 normal volunteers showed an average decline in creatinine clearance of 0.75 mL/min/year [21]. Bolignano et al. [22] analyzed three cohorts and nine cross-sectional studies on age-related decline in kidney function conducted between 1950 and 2012, finding that the decline in GFR ranged from 0.4 to 2.6 mL/min/year. Waas et al. [23] analyzed factors influencing the absolute and relative changes in estimated GFR (eGFR) over a 5-year period in 12,381 participants of the Gutenberg Health Study, confirming a median annual eGFR loss of approximately 1 mL/min/1.73 m^2^. About half of the population aged 70 and older have an eGFR < 60 mL/min/1.73 m^2^ [2, 24]. The Kidney Disease: Improving Global Outcomes organization defines CKD as a slow decline in GFR to below 60 mL/min/1.73 m^2^ or the presence of markers of kidney damage for at least 3 months [17, 25]. Thus, distinguishing renal aging from CKD is important, especially in the elderly. GFR is a comprehensive indicator of kidney function, while albuminuria is a key marker of kidney dysfunction or damage. Detecting albuminuria is crucial for differentiating normal age-related GFR decline from CKD. However, distinguishing aging from CKD in the absence of proteinuria remains challenging.

In clinical practice, GFR is commonly estimated using formulas (eGFR) due to the cumbersome nature of direct measurement. The CKD-EPI 2021 equation is recommended for eGFR as it performs well across age groups and excludes race coefficient, with less bias from age-related muscle mass changes. Cystatin C serves as a confirmatory marker when creatinine-based estimates are unreliable (e.g. in frail or sarcopenic elderly). Creatinine-based eGFR is simple but may overestimate GFR in frail elderly or underestimate it in those with high muscle mass, due to age-related decreased creatinine production and increased tubular secretion. Cystatin C, less influenced by muscle mass, provides more accurate GFR estimates, especially in early-stage renal dysfunction [26, 27].

### Decline in renal blood flow

Davies et al. reported that renal blood flow (RBF) in normal individuals is maintained at approximately 600 mL/min and begins to decline by about 10% per decade starting around age 40 [28]. RBF can be assessed by measuring the clearance of para-aminohippurate (PAH). The average PAH clearance is 649 mL/min between the ages of 40 and 50 but decreases to an average of 289 mL/min between the ages of 90 and 100 [29]. Furthermore, age-related alterations in the renal response to vasoactive substances may also contribute to the decline in RBF [30, 31]. Non-invasive alternatives such as arterial spin labeling-magnetic resonance imaging (ASL-MRI) and contrast-enhanced ultrasound are promising but require further validation and are not yet widely available.

### Decline in renal tubular function

Beyond glomerular filtration, aging also impacts the tubular transport functions for water and electrolytes. Multiple studies have reported a progressive decline in renal tubular function with advancing age, including reduced sodium reabsorption and diminished potassium excretion [2, 32, 33]. Subjects older than 60 years exhibit a decreased ability to reduce sodium excretion following sodium restriction. Conversely, older individuals receiving a sodium load fail to increase sodium excretion appropriately, leading to water and sodium retention. Animal studies indicate that aged rats have a reduced efficiency of renal potassium excretion when fed a high-potassium diet. Older adults are also more prone to acid-base imbalances. The age-related decline in GFR reduces the kidney’s capacity to buffer metabolic changes and excrete excess H^+^ and ammonium. The ability of the renal tubules to concentrate and dilute urine is another crucial indicator of renal function. In the aforementioned Baltimore longitudinal study, compared to two younger groups (20–39 and 40–59 years old) subjected to a 12-h water deprivation test, subjects aged 60–79 showed a 20% reduction in maximum urine osmolality and a 50% decrease in solute conservation capacity [31, 34]. Given its high sensitivity to age-related medullary changes, maximum urine osmolality after water deprivation (or vasopressin stimulation) is recommended as a practical indicator of renal tubular aging, particularly in research settings.

### Endocrine dysfunction

The renin–angiotensin–aldosterone system is a critical system for maintaining body fluid balance and blood pressure homeostasis. Renin, secreted by the juxtaglomerular cells of the kidney, catalyzes the conversion of angiotensinogen in the plasma to angiotensin I (AT-I). AT-I is then converted to angiotensin II (AT-II) by angiotensin-converting enzymes. AT-II promotes vasoconstriction and the release of aldosterone. The aging kidney exhibits reduced synthesis and release of renin, leading to decreased plasma renin activity (PRA) and aldosterone levels [35, 36]. A small cohort study showed that PRA declines with age, particularly in individuals over 40 years old [37]. Studies in aged rats have also confirmed that both plasma renin levels and activity decrease with age [38, 39]. Research by Corman et al. [40] found that the renin content in kidney tissue of 30-month-old female Wistar Albino Glaxo from Rijswijk (WAG/Rij) rats was half that of 10-month-old rats. A report by Jung et al. [41] confirmed an impaired renin release rate in aged Sprague–Dawley rats.

1,25-Dihydroxyvitamin D_3_ (1,25(OH)_2_D_3_) is the active form of vitamin D and plays a vital role in maintaining calcium homeostasis. Vitamin D, obtained from diet or skin synthesis, is hydroxylated in the liver to 25-hydroxyvitamin D_3_, then converted in the kidneys by 1α-hydroxylase to 1,25-dihydroxyvitamin D_3_, which can be further catalyzed by renal 24-hydroxylase to the inactive 24,25(OH)_2_D_3_. Results from a small-scale human study and animal experiments indicate that serum 1,25(OH)_2_D_3_ levels decrease with age, and research has demonstrated an age-related decline in the kidney’s ability to produce 1,25(OH)_2_D_3_ [42, 43]. Furthermore, animal studies have found that the mRNA expression of renal 24-hydroxylase (*CYP24A1*) is upregulated with age, which is another reason for the decrease in 1,25(OH)_2_D_3_ in aging [44].

### Recommendations

1. GFR is a validated functional biomarker suggestive of renal aging (Level A evidence, Class I recommendation). GFR can be assessed via eGFR based on serum creatinine levels.
2. RBF is a validated functional biomarker suggestive of renal aging (Level A evidence, Class I recommendation). The clinical gold standard for measurement is radionuclide determination of RBF; however, due to its radioactive nature, it is not commonly used. Alternatively, the clearance of specific substances, such as PAH, can be utilized for assessment.
3. Renal capacity for urine concentration and dilution is a validated functional biomarker suggestive of renal aging (Level A evidence, Class I recommendation). This can be measured using concentration and dilution tests.
4. PRA is a validated functional biomarker suggestive of renal aging (Level B evidence, Class IIa recommendation). PRA can be measured using methods such as enzyme-linked immunosorbent assay (ELISA) or radioimmunoassay.

## Structural characteristics and assessment of renal aging

### Reduction in renal volume and weight

Population-based surveys in China indicate that the average adult kidney measures approximately 10–12 cm in length and 5–6 cm in width. In elderly individuals, the longitudinal diameter decreases to 9.2–11.8 cm. Between the ages of 70 and 80, renal weight declines by 20%–30%, and volume decreases by approximately 20% compared to normal adults [45, 46]. A study involving 1852 adults that utilized MRI estimated that total renal volume decreases by approximately 16 cm³ every decade, with most loss occurring after age 60 [47]. Similarly, a cohort of 1334 living kidney donors showed through CT scanning that renal volume declines by approximately 22 cm^3^ per decade after age 50. Contrast-enhanced CT angiography allows for the separate quantification of cortical and medullary volumes; the cortex accounts for 73% of total renal volume, while the medulla comprises 27%. With aging, cortical volume and thickness decrease, whereas medullary volume relatively increases [48]. These changes, occurring in the absence of identifiable pathology, are defined as physiological renal atrophy.

Clinically, renal size is routinely assessed through ultrasonography—a non-invasive imaging modality that employs high-frequency sound waves to generate images of the kidneys and perirenal structures, providing information on size, morphology, internal architecture, and blood flow.

### Nephrosclerosis

Nephrosclerosis is characterized histopathologically by arterio-/arterial sclerosis, focal global glomerulosclerosis, tubular atrophy, and interstitial fibrosis. Changes in the arterial wall, such as fibrous intimal thickening and/or hyaline degeneration, lead to ischemia that causes focal glomerulosclerosis, adjacent tubular atrophy, and interstitial fibrosis [49]. Diagnosis necessitates a percutaneous renal biopsy. Although nephrosclerosis is closely associated with hypertension, it is also observed in normotensive, otherwise healthy adults. Among 1203 living donors, the prevalence of nephrosclerosis increased from 2.7% in individuals aged 18–29 to 73% in those aged 70–77. Age-related nephrosclerosis can be challenging to distinguish from chronic changes secondary to specific renal diseases [50, 51]. In a study of 1847 normotensive donors, the 95th percentile threshold for sclerotic glomeruli per 20-glomerulus biopsy was 1.0 at age 20 and 5.5 at age 70. Biopsies that exceeded this threshold exhibited a 3-fold higher frequency of Bowman’s capsule thickening, pericapsular fibrosis, and capillary tuft collapse in non-sclerotic glomeruli, indicative of ischemic injury [52]. In addition to glomerulosclerosis, tubular atrophy and interstitial fibrosis are prominent age-related alterations [53–55]; renal surface granularity also increases with age [49, 56].

### Decline in nephron number and compensatory hypertrophy

At birth, the two kidneys contain approximately 2 million nephrons, a number that is generally maintained until the ages of 18–29. Data from the Mayo Clinic demonstrate a mean loss of 6207 nephrons per year after age 30, with nearly 50% lost by ages 70–75 [51]. Cross-sectional studies indicate that renal function begins to decline after age 40, accelerates after 50, and from age 60 onward, and an additional 6000–6500 nephrons are lost annually. The remaining nephrons undergo compensatory hypertrophy to mitigate the initial decline in GFR [51, 57]. Residual glomerular hyperfiltration and intraglomerular hypertension up-regulate transforming growth factor-α and epidermal growth factor receptor expression, further promoting hypertrophy. When hyperfiltration exceeds a critical threshold, podocyte detachment, focal segmental glomerulosclerosis, and global glomerulosclerosis ensue, leading to nephron loss, additional hyperfiltration of surviving nephrons, and a vicious cycle of progressive deterioration.

### Recommendations

1. Reduction in renal volume is a validated imaging biomarker suggestive of renal aging (Level A evidence, Class IIa recommendation).
2. Nephrosclerosis can be utilized as a histopathological biomarker of renal aging (Level B evidence, Class IIb recommendation).
3. Decreased nephron number with compensatory hypertrophy is a histopathological biomarker consistent with renal aging (Level B evidence, Class IIb recommendation).

## Humoral biomarkers

### Serum protein biomarkers

#### Klotho

Klotho, a well-established “longevity protein” synthesized mainly in kidneys, plays a critical role in aging and numerous diseases. Its concentration in serum has been demonstrated to diminish in aged mice [58]. Multiple clinical studies have illustrated a progressive decline in serum Klotho level with the advancing age. Similarly, significantly reduced Klotho levels have also been observed in patients with CKD. A cross-sectional study recruiting 13,589 participants further reported an inverse correlation of serum Klotho level with age, alongside a positive association with the incidence of CKD [59]. In a separate study, domestic scholars revealed that plasma Klotho closely related to the kidney function decline in the elderly [60]. In the mechanistic studies using animal models, the reduced circulating Klotho could lead to a dysregulated elevation of FGF23 [61]. It has been known that the increased FGF23 are linked to aging, and the subsequent progressively impaired kidney function and nephron loss, thereby augmenting the phosphate excretory load of the residual nephrons [62]. Taken together, Klotho is a promising serum protein biomarker for renal aging. However, soluble Klotho (sKlotho) levels are affected by circadian rhythm, exercise, diet, and acute stress, resulting in high variability. Its narrow dynamic range and large fluctuations limit precision as an aging marker, warranting caution when interpreting sKlotho in renal aging.

#### N-terminal Pro-B-type natriuretic peptide

N-terminal Pro-B-type natriuretic peptide (NT-proBNP) originates from ventricular myocytes. Studies have demonstrated that plasma NT-proBNP levels were significantly increased in patients with CKD, and its predictive value for individual aging has gained considerable interest in recent years. Fabbian et al. [63] found that plasma NT-proBNP level was inversely correlated with eGFR in the elderly. Therefore, NT-proBNP can be regarded as a relevant serum protein biomarker for renal aging.

#### Senescence-associated secretory phenotype

Senescent cells still survive, but their metabolic activities and the corresponding gene expression undergo dramatic changes, forming a complex group of senescence-associated secretory phenotypes (SASPs). Transforming growth factor-β (TGF-β) and tissue inhibitors of metalloproteinases 1 (TIMP1) are important SASPs. A community-based cohort study of the elderly populations revealed that increased plasma TGF-β level was significantly associated with decreased eGFR [64]. In addition, through analyzing plasma proteomics data from 240 healthy adults (aged 22–93 years), Tanaka et al. [65] found that plasma TIMP1 may be involved in aging-related kidney impairment. Therefore, TGF-β and TIMP1 can be considered as potential serum protein markers associated with renal aging. Furthermore, SASPs also include inflammatory factors (e.g., IL-1α, IL-1β, IL-6, and IL-8), growth factors (e.g., HGF and GM-CSF), and chemokines (e.g., CXCL1, CXCL3, and CXCL10). Studies utilizing naturally aged animal models have confirmed that the concentrations of multiple SASPs significantly increased in senescent kidneys [66], which may serve as candidate serum protein biomarkers for renal aging. However, relevant population-based studies are currently lacking. It should be noted that these factors are non-specific indicators of systemic aging (inflammaging) and are influenced by multiple extra-renal conditions; therefore, they are not recommended as standalone markers for renal aging.

#### Cyclin-dependent kinase inhibitor

p21-Waf1/Cip1 and p16INK4A, the common biomarkers of cellular senescence, belong to the family of cyclin-dependent kinase inhibitors. Johnson and Zager [67] found that plasma p21-Waf1/Cip1 may be a potential biomarker for renal aging in mice, which requires further validation in population-based studies. Through inhibiting the activity of cyclin-dependent kinases 4 and 6, p16INK4A prevents the phosphorylation of the retinoblastoma protein (Rb), thereby blocking cells from progressing from the G_1_ phase to the S phase of the cell cycle. During renal aging, the upregulated p16INK4A may induce cell cycle arrest and impair proliferative capacity in kidney cells, which in turn accelerates the aging process [68, 69]. In summary, p21-Waf1/Cip1 and p16INK4A could be considered as serum protein biomarkers for renal aging.

### Serum metabolic biomarkers

#### Amino acids and their metabolites

Homocysteine (Hcy), a sulfur-containing amino acid and methionine metabolite, is elevated in aging and independently predicts kidney disease. A cohort study involving 1135 elderly individuals revealed a significant positive correlation between kidney function decline and plasma Hcy levels [70]. A 6-year Chinese study (*n *= 1765, mean 58 years) linked glutamine and phenylalanine to annual eGFR decline [71]. Elderly metabolomics studies also implicate methionine, histidine, arginine, asparagine, threonine, and glycine as potential renal aging biomarkers [72–74].

Amino acid metabolites also have predictive value. Advanced glycation end products (AGEs) are implicated in aging [75]. Supporting this, an Italian cohort study enrolling participants aged 65 years and above showed that plasma concentration of the key AGE carboxymethyl-lysine (CML) significantly correlated with the onset of CKD, identifying CML as an independent risk factor for the deterioration of kidney function in the elderly [76].

#### Lipids and related derivatives

In a 2022 cohort of 1910 American Indians (mean age: 40), Zeng et al. [77] found that longitudinal perturbations in the plasma lipidome—including triglycerides, phosphatidylcholines, phosphatidylethanolamines, sphingomyelins, fatty acids, and acylcarnitines—correlated with kidney function decline, independent of clinical covariates and baseline eGFR. In 2018, Wang et al. identified 10 plasma acylcarnitines (C3DC, C5OH, C8, C10, C14:1OH, C16:2, C18, C18:2, C20, C20:4) positively associated with age-related eGFR decline [71]. In 2024, a large-scale cohort of 91,532 individuals (mean age: 55) using nuclear magnetic resonance (NMR) spectroscopy showed that increased very low density lipoprotein (VLDL) particles predicted new-onset CKD risk, while high-density lipoprotein (HDL) appeared protective [78]. Nonetheless, their potential to predict renal aging has yet to be verified.

#### Gut microbiota-derived metabolites

Uremic toxins, harmful metabolites that accumulate as kidney function declines, are biological indicators of renal impairment. Many are generated by gut microbial metabolism and termed gut-derived uremic toxins, including indoxyl sulfate (IS), and indole acetic acid (IAA). These toxins damage kidney cells and drive glomerulosclerosis, tubulointerstitial inflammation, and fibrosis, contributing to CKD progression [79–81]. Emerging evidence suggests that IS and PC may induce cellular senescence in tubular or endothelial cells, leading to kidney function decline [82].

Gut-derived uremic toxins are also associated with age-related kidney function decline. Sun et al. [83] studied 151 subjects from a Guangxi longevity village and found that serum levels of p-cresol , *N*-phenylacetyl-l-glutamine, 2-hydroxyindole, and 4-aminohippurate increased with age and correlated with creatinine, urea nitrogen, and cystatin C. Notably, fecal microbiota transplantation from octogenarians to young mice increased systemic levels of these nephrotoxic metabolites.

Hydrogen sulfide (H_2_S), a gut microbiota-derived signaling molecule, decreases with age. Animal studies show that H_2_S administration mitigates age-related kidney injury in mice [84], and a meta-analysis reported reduced serum H_2_S levels in kidney disease patients [85].

#### Other metabolites

Malondialdehyde (MDA), an end product of lipid peroxidation, is involved in oxidative stress and aging. Analysis of 2169 Chinese Han adults showed that high plasma MDA may independently predict kidney function decline in the elderly, suggesting its potential as a serum metabolite biomarker for renal aging [86]. Yamaguchi et al. [72] found significant correlations between plasma acylcarnitines, hexoses, and choline with age and declining eGFR in an elderly cohort. These metabolites are candidate blood biomarkers for renal aging, but verification in larger cohorts is warranted.

### Recommendations

#### Serum protein biomarkers

1. Klotho may serve as a serum protein biomarker for predicting renal aging, with its downregulation suggesting a potential age-related kidney function decline (Level A evidence, Class I recommendation).
2. TGF-β could be considered as a candidate serum protein biomarker for predicting renal aging, as its elevation may indicate renal aging (Level B evidence, Class IIa recommendation).
3. Increased NT-proBNP may suggest renal aging. However, large-scale population-based cohorts are warranted (Level C evidence, Class IIb recommendation).
4. TIMP1, especially when increased, exhibit a potential as a biomarker for predicting renal aging. Nevertheless, further validation in large-scale population-based cohorts is needed (Level C evidence, Class IIb recommendation).
5. Increased concentrations of various types of SASPs represent potential serum protein biomarkers for renal aging, but this awaits confirmation in population-based studies (Level C evidence, Class IIb recommendation).
6. p21-Waf1/Cip1 and p16INK4A are potential serum protein biomarkers for predicting renal aging. Specifically, increased serum concentrations of p21-Waf1/Cip1 and p16INK4A may suggest renal aging, though this requires further validation in population-based studies (Level C evidence, Class IIb recommendation).

#### Serum metabolic biomarkers

1. Elevated blood homocysteine, a routine clinical parameter, is a well-established indicator of renal aging. Therefore, it can be considered a serum metabolic biomarker for predicting renal aging (Level A evidence, Class I recommendation).
2. Elevated serum concentrations of glutamine and phenylalanine are associated with a decline in eGFR and potential renal aging, but their use as serum metabolic biomarkers is hindered by high testing costs (Level A evidence, Class IIa recommendation).
3. Elevated blood levels of CML may serve as a serum metabolic biomarker for predicting renal aging, with increased concentrations suggesting a potential age-related kidney function decline. However, its application is limited by high testing costs (Level A evidence, Class I recommendation).
4. Elevated serum concentrations of specific acylcarnitines (C3DC, C5OH, C8, C10, C14:1OH, C16:2, C18, C18:2, C20, and C20:4) are indicative of renal aging and may be considered relevant metabolic biomarkers. These acylcarnitines can be measured by tandem mass spectrometry, albeit at a relatively high cost (Level B evidence, Class IIb recommendation).
5. Serum HDL and VLDL, as part of routine and low-cost clinical lipid panels, are potential indicators of renal aging, that decreased HDL and increased VLDL both suggest renal aging (Level A evidence, Class I recommendation).
6. The gut-derived uremic toxins IS and IAA are candidate blood biomarkers for predicting renal aging, as their elevated concentrations are associated with kidney function decline. These substances can be measured by high-performance liquid chromatography (HPLC) with fluorescence detection at a moderate cost (Level C evidence, Class IIb recommendation).
7. Elevated serum gut-derived uremic toxins p-cresol , *N*-phenylacetyl-l-glutamine, 2-hydroxyindole, and 4-aminohippurate are indicative of renal aging and may serve as serum metabolic biomarkers for its prediction. These substances can be detected using tandem mass spectrometry, though the associated costs are high (Level B evidence, Class IIa recommendation).
8. The gut-derived uremic toxin H_2_S is a potential serum metabolic biomarker for predicting renal aging, with decreased levels suggesting an age-related kidney function decline. H_2_S can be measured by HPLC with fluorescence detection at a moderate cost (Level A evidence, Class I recommendation).
9. Elevated serum MDA suggests renal aging and may serve as a candidate serum metabolic biomarker for its prediction. MDA is a parameter available in clinical testing (Level A evidence, Class I recommendation).

## Urinary biomarkers

### Urinary podocin mRNA

Podocin is a key slit diaphragm protein specifically expressed in glomerular podocytes. During renal aging, progressive podocyte depletion and reduced podocyte density lead to age-associated glomerulosclerosis. Urinary podocin mRNA, derived from detached podocytes in urine sediment, serves as a non-invasive indicator of podocyte injury and loss. Hodgin et al. demonstrated that older individuals exhibit a significantly higher urinary podocin mRNA-to-creatinine ratio compared with younger individuals, reflecting increased podocyte detachment rate with advancing age [55]. Thus, urinary podocin mRNA level may serve as a biomarker of glomerular aging.

### Urine protein marker

α-Klotho is a crucial anti-aging protein primarily expressed in the kidneys, which is the main source of α-Klotho in serum and urine, declining with aging. Furthermore, insufficient Klotho caused by aging kidneys accelerates the development of age-related phenotypes. Transplanting senescent cells into young mice results in reduced α-Klotho secretion in urine. Selective elimination of senescent cells in aged mice through gene editing and pharmacological approaches increases α-Klotho protein in kidneys, brains, and urine. These findings collectively suggest that urine α-Klotho levels may serve as a biomarker for kidney aging [87]. p21 is upregulated in renal tubules in AKI, leading to the degenerative cell senescence/“aging” process. In mice with ischemia-reperfusion-induced AKI, p21 expression in renal cortex was upregulated, accompanied by significant elevation of p21 levels in plasma and urine. Interestingly, aging was associated with progressive renal cortical p21 expression, which correlated with increased plasma p21; however, urinary p21 was undetectable throughout the aging process. Therefore, whether urine p21 can serve as biomarker of aging kidney requires further clinical investigation [67].

### Urinary metabolites

Metabolic changes associated with aging in organs can also be detected in urine. An elegant research examined the levels of ascorbic acid (AA) in 14 organ tissues, plasma, and urine of male C57BL/6 mice and monitored its variations throughout the aging process from 3 to 30 months. They identified a notable decrease in urinary AA levels with the progression of aging, barely detectable at 24 and 30 months. Urinary AA might serve as an indicator of the diminished AA synthesis capacity in multiple organs during the aging process [88]. Furthermore, a study utilizing mass spectrometry data obtained from the urine of aged mice identified 3-methylhistidine, 4-imidazolylacetic acid, o-aminobenzoic acid (vitamin L1), DL-lactate, glucose, glycolic acid, L‑threonate acid, *N*-acetyl-l-glutamic acid, and urate as potential biomarkers associated with age-related kidney dysfunction [89]. Consequently, metabolic biomarkers in urine may undergo alterations during the aging process. Nevertheless, additional population-based studies are required to validate these findings. Furthermore, owing to the elevated technical prerequisites and costs associated with metabolite detection, clinical implementation remains a challenge.

### Urine extracellular vesicles

Renal parenchymal cells exhibit dynamic secretion of extracellular vesicles (EVs) into urine. The detection of surface proteins or contents of urinary EVs may possess substantial significance in reflecting the cellular state of aging kidneys. A clinical investigation encompassing patients with primary hypertension, renovascular hypertension, and healthy volunteers was conducted. Urinary EVs were collected for imaging flow cytometry analysis of aging cell markers p16 and tubular markers at different stages. The results revealed an elevated quantity of p16^+^ EVs originating from proximal tubules in all hypertensive patients. Moreover, in patients with renovascular hypertension, p16^+^ EVs were predominantly derived from the loop of Henle. This implies that urinary EVs may contribute to identification of specific types of aging cells within the kidneys [90]. Most of previous studies suggest that the secretion of EV increases with aging and in age-related diseases; however, some inconsistent data may attribute to the variations in cell culture scale and aging-inducing factors. Urinary EVs from female were higher than those of males as evaluated by EVs per milligram of creatinine or EVs per milliliter of urine. Besides, the quantity of EVs released from juxtaglomerular cells and podocytes declines with aging [91]. Increasing studies suggested that the cargo and functions of EVs change with aging. Senescent cells secrete EVs that encompass age-related proteins, glycosylated proteins, non-coding RNAs, and lipid components, which mirror the characteristics of senescent cells. In comparison with young rats, aged rats demonstrated upregulated levels of miR-21 in urinary EVs [92]. Besides, urinary EVs from elderly spontaneously hypertensive male rats were abundant in lipid components and were capable of activating epithelial sodium channel (ENaC) channel proteins in the collecting duct cells, thus participating in the temporal regulation of hypertension in aged mice [93]. Nevertheless, research on the contents of EVs derived from kidney senescent cells remains limited and further investigations are required to validate the potential of EV cargo as a marker of aged kidney.

### Recommendations

1. Podocin mRNA within urine sediment can be a biomarker of renal aging. An upregulated expression of this mRNA suggests the loss of podocytes in the aging kidney. This biomarker can be routinely detected via RT-PCR at a relatively low cost (Level B evidence, Class IIa recommendation).
2. The urinary α-Klotho level can potentially function as a biomarker for renal aging, where reduced levels signify the aging process, especially within the kidneys. It can be detected via ELISA at a reasonable cost (Level C evidence, Class IIb recommendation).
3. Reduced urinary AA levels can potentially function as a biomarker for renal aging, indicating diminished AA synthesis capacity in multiple organs. HPLC can be applied for detection at relatively high costs (Level C evidence, Class IIb recommendation).
4. Elevated levels of the p16^+^ EVs may serve as a urinary biomarker for renal aging. The detection of cellular markers in the p16^+^ EVs by high-sensitivity flow cytometry can facilitate the identification of parent aging cell types in the kidneys. However, additional clinical evidence is still needed (Level C evidence, Class IIb recommendation).

## Conclusion and future perspectives

### Key recommended biomarkers for kidney aging

Based on expert discussions, biomarkers for kidney aging are recommended across three dimensions: function, structure, and body fluids, while also considering the feasibility of their detection technologies for widespread clinical application (Table 2). These biomarkers will require further validation across population cohorts of different age groups in the future.

**Table 2.** Biomarker recommendations for renal aging.

Dimension	Biomarker	Testing method	COR	LOE
**Functional markers**	Glomerular filtration rate (GFR) decreased	Serum creatinine (enzymatic) → eGFR (CKD-EPI)	I	A
	RBF decreased	Para-aminohippurate clearance, radionuclide imaging	I	A
	Renal tubule dysfunction	Concentration and dilution test	I	A
	Renal endocrine dysfunction	ELISA for endocrine factors (renin)	IIa	B
**Structural markers**	Volume and weight decreased	Ultrasound, CT	IIa	A
	Nephrosclerosis	Biopsy	IIb	B
	Nephron number decreased	Biopsy	IIb	B
**Humoral biomarkers: serum protein biomarkers**	Klotho	Plasma/ELISA	I	A
	TGF-β	Plasma/ELISA	IIa	B
	NT-proBNP	Plasma/ELISA	IIb	C
	TIMP1	Plasma/ELISA	IIb	C
	SASPs	Plasma/ELISA	IIb	C
	p21-Waf1/Cip1 and p16INK4A	Plasma/ELISA	IIb	C
**Humoral biomarkers: serum metabolic biomarkers**	Homocysteine	Plasma/ELISA	I	A
	Glutamine and phenylalanine	Plasma/ELISA	IIa	A
	Carboxymethyl-lysine (CML)	Plasma/ELISA	I	A
	Acylcarnitines (C3DC, C5OH, C8, C10, C14:1OH, C16:2, C18, C18:2, C20, and C20:4)	Plasma/ELISA	IIb	B
	HDL and VLDL	Plasma/ELISA	I	A
	Indoxyl sulfate (IS) and indole acetic acid (IAA)	Plasma/ELISA	IIb	C
	p-cresol, *N*-phenylacetyl-l-glutamine, 2-hydroxyindole, and 4-aminohippurate	Plasma/ELISA	IIa	B
	H_2_S	Plasma/ELISA	I	A
	MDA	Plasma/ELISA	I	A
**Urinary biomarkers**	Podocin mRNA	RT-PCR	IIa	B
	α-Klotho	ELISA	IIb	C
	Ascorbic acid	HPLC	IIb	C
	p16^+^ extracellular vesicles	High-sensitivity flow cytometry	IIb	C

### Roadmap for research on kidney aging biomarkers in China

1. Establish Multi-Center Aging Cohorts and Methodologies in China to develop detection technologies, integrate clinical and fundamental research, validate kidney aging biomarkers for the Chinese population, identify aging inflection points, and define personalized intervention windows.
2. Develop AI-based kidney biological age assessment and disease prediction models to construct a measurement system and related predictive tools.
3. Promote deep integration of industry, academia, and research to accelerate translational application. Establishing a kidney aging biomarker system will advance kidney disease research and improve kidney health among the elderly in China.
